# Evaluation of the effect of coenzyme Q10 supplementation along with scaling and root planing (SRP) on periodontal and gingival indices in controlled diabetic patients

**DOI:** 10.34172/japid.2022.003

**Published:** 2022-03-13

**Authors:** Shima Ghasemi, Zeinab Torab, Adileh Shirmohammadi, Amirreza Babaloo, Reza Johari, Farrokh Farhadi, Majid Mobasseri, Hamidreza Mohammadi

**Affiliations:** ^1^Department of Prosthodontics, Faculty of Dentistry, Tabriz University of Medical Sciences, Tabriz, Iran; ^2^Department of Pediatric Dentistry, Faculty of Dentistry, Tabriz University of Medical Sciences, Tabriz, Iran; ^3^Department of Periodontics, Faculty of Dentistry, Tabriz University of Medical Sciences, Tabriz, Iran; ^4^Private practic, Tabriz, Iran; ^5^Department of Oral and Maxillofacial Surgery, Faculty of Dentistry, Tabriz University of Medical Sciences, Tabriz, Iran; ^6^Department of Internal Medicine, School of Medicine, Endocrine Research Center, Imam Reza Medical Research & Training Hospital, Tabriz University of Medical Sciences, Tabriz, Iran

**Keywords:** Bleeding on probing, clinical attachment level, coenzyme Q10, periodontitis, plaque index, probing depth, gingival index

## Abstract

**Background:**

This clinical trial evaluated the effect of coenzyme Q10 supplementation along with scaling and root planing (SRP) on periodontal and gingival indices in controlled diabetic patients.

**Methods:**

Forty-two diabetic patients (controlled type), referred to the Department of Periodontics with chronic periodontitis and eligible for the study, were included in the study. Patients suffering from chronic periodontitis with a probing pocket depth of ≥5 mm in different quadrants of the oral cavity with radiographic evidence of bone loss were included in the present randomized, double-masked, placebo-controlled clinical trial. The subjects were instructed to take one capsule of coenzyme Q10 or a placebo every day for 30 days following SRP. Clinical parameters, i.e., plaque index (PI), gingival index (GI), bleeding on probing (BOP), clinical attachment level (CAL), and probing pocket depth (PPD), were recorded at baseline and four weeks after treatment by two masked and calibrated examiners. The study results were reported as (mean ± standard deviations) and frequencies (percentages).

**Results:**

One month after the intervention, PPD, CAL, BOP, and PI indices in the intervention group were significantly lower than those in the control group. One month after the intervention, the GI was similar in both groups. A significant decrease was observed in the GI in both groups after the intervention.

**Conclusion:**

The results of the present study showed that Q10 orally with scaling and root planing in patients with controlled diabetes with chronic periodontitis might accelerate the treatment process and significantly reduce the pocket depth.

## Introduction

 Periodontal disease is one of the most important and common diseases of the oral cavity. Periodontitis is an inflammatory response of periodontal tissues to plaque microorganisms that can cause tissue damage and disease progression by increasing the pocket depth, reducing the supporting tissues of the teeth, bleeding, and causing pus discharge from the gingival tissues, abscesses, and pain.^1^ Common indicators for measuring periodontitis currently include pocket depth, bleeding on probing, plaque index, and clinical attachment loss, but no laboratory tests are currently available to determine patients with periodontitis that are not routinely treated.^2^ It is a multifactorial disease initiated by microbial plaque, but its spread and severity depend on environmental factors, acquired diseases, and genetic predisposition. Destruction of tooth-supporting tissues and loosening of teeth with their eventual loss are the most important complications of this disease.^3^In a susceptible host, microbial pathogens can destroy periodontal tissues by stimulating the release of host inflammatory enzymes and cytokines.

 Molecular assays consider the mediation of concomitant inflammatory responses, of which free radicals and reactive oxygen species (ROS) are the most important. Periodontal pathogens can lead to ROS overproduction, destroying periodontal cells and collagen structure. When antioxidants destroy ROS, collagen breakdown can be prevented. Coenzyme Q10 (Co Q10) acts as an endogenous antioxidant that increases the concentration of this coenzyme in gingival diseases and effectively inhibits advanced periodontal inflammation.^4^ Co Q10 was discovered in 1957 in cardiac mitochondria. Its chemical structure and synthesis were completed in 1958. This coenzyme is known as ubiquinone because of its widespread presence in nature and its quinone structure (similar to vitamin K).^5^

 Bacteria possess several structurally different quinones, among which ubiquinone (UQ), menaquinone (MK), and demethylmenaquinone (DMK) are the most common. These quinones are found in the cytoplasmic membrane, where they participate as electron carriers in respiration and disulfide bond formation. UQ participates in aerobic respiration, whereas MK and DMK have roles in anaerobic respiration. UQ molecules are classified based on the length (n) of their isoprenoid side chain (UQ-n). For example, the main UQ species in humans is UQ-10; in rodents, it is UQ-9; in Escherichia coli, it is UQ-8; and in Saccharomyces cerevisiae, it is UQ-6 in varying amounts.^6^ Coenzyme Q9 is the predominant form in relatively short-lived species such as rats and mice, whereas in humans and other long-lived mammals, the major homolog is coenzyme Q10.

 Numerous experimental and clinical studies have shown the importance of Co Q10 in lowering blood pressure. Only one study has evaluated the effect of long-term supplementation with this coenzyme on type II diabetic patients.^6^ The gold standard in the treatment of periodontal disease is the first phase of treatment, i.e., scaling and root planing and oral hygiene instructions. Non-surgical mechanical treatment reduces inflammation and pocket depth and increases clinical attachment levels.^7^Scaling and root planing alone cannot completely remove plaque and calculus due to microbial invasion of soft tissues. Also, areas that are not anatomically accessible, such as furcation and root concavities, are not thoroughly cleaned, especially in deep pockets. The remaining plaque and calculus can cause treatment failure.^8,9^

 Manthena et al^10^ studied the effects of oral administration of Co Q10 as an adjuvant on periodontal health indicators and showed that inflammatory markers decreased relative to baseline values in both intervention and control groups in the first and third months. Although the plaque index and probing depth values at both intervals did not show a significant difference, the two groups were significantly different in the first and third months in terms of gingivitis. This study showed that Co Q10 administration reduced gingival inflammatory markers. In diabetic patients, the amount of Co Q10 received from their diet is reduced due to their special diet.^11^

 Since no studies have evaluated the effects of Co Q10 and SRP on controlled diabetic patients and the results of various studies are inconsistent, the present study examined the effects of Co Q10 administration and the simultaneous effects of Co Q10 and SRP administration on periodontal and gingival indices in controlled diabetic patients.

## Methods

 All the rights of human subjects were observed in the present randomized, double-blind, placebo-controlled clinical trial. The Ethics Committee of Tabriz University of Medical Sciences approved this study under the code 49867. The study protocol conforms to the ethical guidelines of the 1975 Declaration of Helsinki as revised in 2013. Forty-two diabetic patients (controlled type) referred to the Department of Periodontics, Faculty of Dentistry, Tabriz University of Medical Sciences, with chronic periodontitis, eligible to be included in the study were evaluated. Systemically healthy patients in the 29–59 age group of both genders (mean age = 33.8 years) diagnosed with chronic periodontitis by their clinical and radiographic findings were included in the study. Written and verbal consent was obtained from the subjects recruited for the study. Patients suffering from chronic periodontitis, with a probing pocket depth of ≥5 mm in different quadrants (having a minimum of six permanent teeth in each quadrant) of the oral cavity with radiographic evidence of bone loss were included in the study.^12^ Patients with a history of any systemic diseases, any apparent oral infection like herpes or candidiasis, patients who had taken antibiotic therapy in the past three months or undergone any periodontal therapy in the past six months, smokers, pregnant women, and lactating mothers were excluded from the study.

 Randomization was performed by placing 21 red (controls) and 21 green (test) marbles in a bag, from which the patient selected one. Co Q10 supplement capsules containing 100 mg of Co Q10 were given to the test group, while placebo capsules were administered to controls. Two calibrated dental examiners recorded all measurements. The dental examiners remained masked to the study treatments of all the patients throughout the study period. All the patients underwent SRP in the study quadrant by the same clinician. Patients were given a bottle containing 50 test (Co Q10) or control (placebo) capsules immediately after treatment and instructed to take one capsule each morning until the 30-day postoperative appointment.

**Figure 1:**
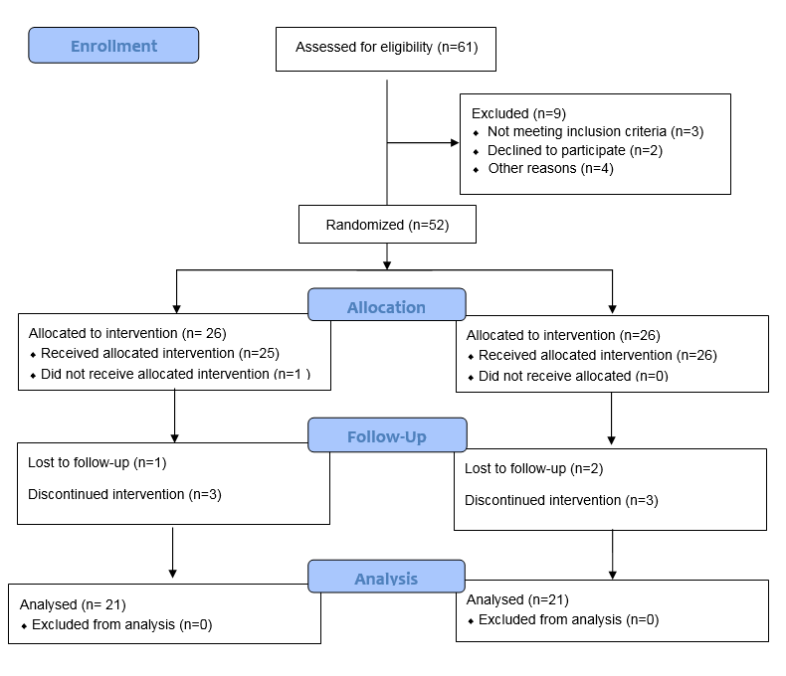
Consolidated Standards of Reporting Trials Diagram.

 The clinical parameters evaluated in this study included clinical attachment level (CAL), measured from the cementoenamel junction or a fixed reference point to the depth of the periodontal pocket; pocket probing depth (PPD), measured from the gingival margin to the probable base of the periodontal pocket; and bleeding on probing (BOP), recorded at each site as present or absent. In addition, the gingival index (GI) and plaque index (PI) scores were evaluated.^11^ CAL was used to estimate the actual gains of periodontal attachment levels.^13,14^ GI was used to record the degree of soft tissue inflammation during soft tissue healing. PI was used to monitor improvements in patients’ oral hygiene and GI.^11^ All measurements were performed using a University of North Carolina probe at six sites per tooth.^15^ All the clinical parameters were recorded at baseline and four weeks after treatment by two masked and calibrated examiners. To improve the reporting of the randomized clinical trial, we followed the Consolidated Standards of Reporting Trials Diagram (Figure 1).

## Data analysis

 The study results were reported as (means± standard deviations) and frequencies (percentages). T-test was used to compare PPD, CAL, BOP, GI, and PI between the two groups. SPSS 17 was used for data analysis. In this study, the probability value of P<0.05 was considered statistically significant.

## Results

 In this clinical trial, 42 patients with controlled diabetes and chronic periodontitis were studied in two intervention groups (SRP treatment and Q10 administration) and a control group (SRP treatment alone).

 The present study included 27 females and 15 males with a mean age of 33.8±8.93 years. There was no statistically significant difference in clinical parameters (PPD, CAL, BOP, and PI) between the two groups at baseline.

Table 1 shows the HbA1C and FBS values of patients. Acceptable levels of fasting blood sugar (FBS) and long-term glucose (HbA1C) according to the American Clinical Endocrine Society are 70-130 and 7>, respectively.

**Table 1:** Fasting and long-term blood sugar levels in controlled diabetic patient

**Group**	FBS* **(Normal Range 70-130)**		**HbA1C** **(Normal rage**<**7**)	
	**Mean**	**SD**	**Mean**	**SD**
**Intervention Group (n=42)**	12.05	12.65	6.78	0.58
**Control Group (n=42)**	10.5	19.71	6.85	0.47
**P-value** ^#^	0.334		0.203	

#P-value: independent t-test *Fasting Blood Sugar

 Fasting blood sugar (FBS) and hemoglobin glycate or long-term glucose (HbA1C) levels were similar in both groups.

Table 2 shows that the PPD parameter was similar between the two groups before the intervention. One month after the intervention in both groups, PPD decreased significantly (P<0.001). Also, one month after the intervention, the PPD in the intervention group was significantly lower than in the control group (P<0.001).

**Table 2:** Comparison of PPD, CAL, BOP, and PI indices before and after treatment in intervention and control groups

**Index**	**Group**	**Before Treatment**		**One month after treatment**		**P-value** ^#^
		**Mean**	**SD**	**Mean**	**SD**	
**PPD** ^*^	**Intervention Group (n=21)**	4.26	0.91	2.68	1.03	<0.001
	**Control Group (n=21)**	4.34	0.66	3.32	0.96	<0.001
	**P-value** ^##^	**0.743**		**0.042**		
CAL**	**Intervention Group (n=21)**	3.98	0.87	2.49	1.15	<0.001
	**Control Group (n=21)**	3.90	0.66	3.15	0.85	<0.001
	**P-value** ^##^	**0.781**		**0.041**		
BOP***	**Intervention Group(n=21)**	43.43	7.09	26.33	4.91	<0.001
	**Control Group (n=21)**	45.05	5.77	33.14	5.06	<0.001
	**P-value** ^##^	**0.422**		**0.000**		
PI****	**Intervention Group (n=21)**	50.14	8.27	27.67	6.89	<0.001
	**Control Group (n=21)**	49.71	8.74	32.24	6.70	<0.001
	**P-value** ^##^	**0.871**		**0.035**		

##P-value: independent t-test #P-value: paired-sample t-test *Pocket probing depth **Clinical attachment level ***Bleeding on probing ****Plaque index

 The CAL index was similar between the two groups before the intervention. One month after the intervention in both groups, the CAL decreased significantly (P<0.001). Also, one month after the intervention, the CAL in the intervention group was significantly lower than in the control group (P<0.001).

 The BOP index was similar between the two groups before the intervention. One month after the intervention in both groups, the BOP level decreased significantly (P<0.001). Also, one month after the intervention, BOP in the intervention group was significantly lower than in the control group (P<0.001).

 The PI index was similar between the two groups before the intervention. One month after the intervention in both groups, the PI level decreased significantly (P<0.001). Also, one month after the intervention, the PI level in the intervention group was significantly lower than in the control group (P<0.001).

Table 3 shows that the GI index was similar between the two groups before the intervention. One month after the intervention, the GI was similar in both groups. In both groups, a significant decrease was observed after the intervention.

**Table 3:** Comparison of GI index before and after treatment in the intervention and control groups

**Index**	**Group**	**Before Treatment**		**One month after treatment**		**P-value** ^#^
		**Mean**	**SD**	**Mean**	**SD**	
GI**	**Intervention Group (n=21)**	3.38	0.80	2.41	0.68	<0.001
	**Control Group (n=21)**	3.48	0.81	2.60	0.54	<0.001
	P-value*	0.705		0.618		

*P-value: independent t-test #P-value: paired-sample t-test **Gingival Index

## Discussion

 Chronic periodontal disease leads to the progressive destruction of the supporting tissues of the tooth and pocket formation, bone resorption, or both, leading to tooth loss due to extensive destruction of the alveolar bone.^1^

 Chronic hyperglycemia is a major risk factor for periodontal disease.^16^ Studies show that clinical parameters (PI, BOP, CAL, and PPD) are higher in diabetic patients than in healthy individuals.^17^

 In the present clinical trial, forty-two controlled diabetic patients with chronic periodontitis were studied. Blood tests showed that fasting blood sugar (FBS) and long-term blood sugar (HbA1C) were in the normal range. In both the control group (SRP treatment alone) and intervention group (SRP treatment and oral use of Co Q10), a significant decrease was observed in all indicators of PPD, CAL, BOP, and PI in one month. Also, one month after the intervention, the means of these indicators in the intervention group were significantly lower than in the control group.

 One study reported results similar to the present study. The researchers used Co Q10 dietary supplements to treat chronic periodontitis in patients with type 2 diabetes, along with scaling and root planing and observed that three months after treatment, CAL, GI, and PI decreased significantly in the Co Q10 group compared to the control group (SRP alone).^18^

 The nature of Co Q10 in the rapid uptake and loss of electrons makes it a powerful antioxidant.^19^ This product inhibits lipid peroxidation and protein oxidation by inhibiting the production of peroxyl radicals. The regenerative form of this product effectively regenerates vitamin E from the α-tocopherol radical and prevents the degradation of vitamin E.^19,20^ Studies have shown a close relationship between decreased Co Q10 levels and the incidence of diabetes. Accordingly, one of the proposed strategies to reduce the effects of oxidative stress in diabetes is to take Co Q10 supplementation.^19^

 Manthena et al^10^ evaluated the effects of oral administration of Co Q10 as an adjunct to periodontal health indices and showed a significant difference in gingivitis incidence between the control and intervention groups. This study showed that Co Q10 administration reduced gingival inflammatory markers.

 Akbari et al^21^ showed that taking Co Q10 supplementation increased insulin sensitivity in type 2 diabetic patients and recommended taking this supplement in diabetic patients.

 Pranam et al^22^ used Co Q10 gel in a bag to treat patients with chronic periodontitis and observed a significant difference on the two sides of the oral cavity (intervention and control) in the measured periodontal indices. The researchers suggested that CoQ10 gel in the pocket could be a useful adjunct to non-surgical periodontal treatment.

 In the present study, one month after treatment, the gingival index decreased in both groups. However, one month after treatment, the two groups did not significantly differ in the gingival index.

 The above results are contrary to the present study. In the present study, the study period was one month, considered a short time. Long-term oral administration of Co Q10 may show significant effects on reducing gingivitis. Also, the small sample size, the age of the studied patients, and the duration of diabetes are among the influential factors in the difference between the results of the studies.

## Conclusion

 The results of the present study showed that oral Co Q10 with scaling and root planing in patients with controlled diabetes with chronic periodontitis might accelerate the treatment process and significantly reduce the pocket depths.

## Authors’ contributions

 HM initiated, conceptualized, and supervised the research work. HM, SG, and AS contributed to the design of the study. AB, HM, and AS performed the surgeries. ZT and RJ performed data analysis and interpretation of data. ZT and HM, and MM wrote the manuscript. All authors approved the final manuscript.

## Funding

 This work was supported by the Vice-Chancellor for Research, Faculty of Dentistry, Tabriz University of Medical Sciences, Tabriz, Iran.

## Availability of data

 The raw data from the reported study are available upon request from the corresponding author.

## Ethics approval

 This research was approved by the Research Ethics Committee of the Faculty of Dentistry (IR.TBZMED.REC.1399.442).

## Competing interests

 Dr Adileh Sirmohammadi is the editor-in-chief of the journal, and was blinded to peer review of this paper. The authors declare no other competing interests related to the publication of this work.
